# Nascent Iodide of Silver in the Treatment of Conjunctivitis

**Published:** 1882-02

**Authors:** 


### Nascent Iodide of Silver in the Treatment of Conjunctivitis.

According to Sedan (*Recueil d'Ophthalmologie*, May, 1881). M. Brame, of Tours, was among the first to advocate the use of iodide of silver in conjunctivitis. Dr. S. instituted a series of observations, with this remedy, in Algeria, where cases of chronic conjunctivitis are very numerous, and found it decidedly efficacious both as a prophylactic and in the cure of acute and chronic cases.

Two solutions are requisite for the manufacture of the nascent iodide. One of these is a solution of the iodide of potassium, 3 grm. and 32 ctgr. in 10 grm. of distilled water. The other solution is composed of nitrate of silver, 3 grm. and 56 ctgr. in 10 grm. of distilled water. Both solutions are preserved in bottles made of colored glass. The nascent iodide of silver is prepared by dipping separate glass rods into either solution, withdrawing a drop of each and subsequently mingling them on a piece of porcelain. The resulting precipitate of nascent iodide is placed upon the erected lower lid and thence allowed to diffuse itself over the entire conjunctiva. The iodide thus prepared dissolves very slowly in the ocular fluids, and produces much protracted pain. This inconvenience may be obviated by the employment of glycerine as the menstruum. The border of the lids should be anointed with vaseline to prevent them from becoming adherent. Dr. Sedan had constant, rapid, perfect, and permanent results in more than three hundred cases subjected to the treatment in question. He states that a single application will often cure simple cases, while in ninety-two out of one hundred cases, selected at random, less than four applications were necessary to effect a cure.—*The Medical Record.*

